# Pulverization‐Tolerance and Capacity Recovery of Copper Sulfide for High‐Performance Sodium Storage

**DOI:** 10.1002/advs.201900264

**Published:** 2019-04-26

**Authors:** Jae Yeol Park, Sung Joo Kim, Kanghoon Yim, Kyun Seong Dae, Yonghee Lee, Khoi Phuong Dao, Ji Su Park, Han Beom Jeong, Joon Ha Chang, Hyeon Kook Seo, Chi Won Ahn, Jong Min Yuk

**Affiliations:** ^1^ Department of Materials Science & Engineering Korea Advanced Institute of Science and Technology (KAIST) 291 Daehak‐ro Yuseong‐gu Daejeon 34141 Republic of Korea; ^2^ Platform Technology Laboratory Korea Institute of Energy Research Daejeon 152 Gajeong‐ro Yuseong‐gu 34129 Republic of Korea; ^3^ Global Nanotechnology Development Team National Nano Fab Center (NNFC) 291 Daehak‐ro Yuseong‐gu Daejeon 34141 Republic of Korea

**Keywords:** capacity recovery, pulverization tolerance, semi‐coherent interfaces, sodium ion batteries, transmission electron microscopy

## Abstract

Finding suitable electrode materials is one of the challenges for the commercialization of a sodium ion battery due to its pulverization accompanied by high volume expansion upon sodiation. Here, copper sulfide is suggested as a superior electrode material with high capacity, high rate, and long‐term cyclability owing to its unique conversion reaction mechanism that is pulverization‐tolerant and thus induces the capacity recovery. Such a desirable consequence comes from the combined effect among formation of stable grain boundaries, semi‐coherent boundaries, and solid‐electrolyte interphase layers. The characteristics enable high cyclic stability of a copper sulfide electrode without any need of size and morphological optimization. This work provides a key finding on high‐performance conversion reaction based electrode materials for sodium ion batteries.

## 1 Introduction

Since its first commercialization in 1990s, lithium ion batteries (LIBs) have been widely used for various electrochemical energy storage applications including mobile devices, electric vehicles, and energy storage system (ESS). Due to the surge of their demands, lithium and cobalt prices have gone up almost twice since 2015. As a promising alternative for LIBs, thus, sodium ion batteries (SIBs) have been suggested owing to natural abundance and low cost of sodium, and its similar chemical nature to lithium. Despite their growing interest, however, commercialization of SIBs is far from realization due to the lack of suitable electrode materials.

Recently, conversion and alloying reaction materials, such as Co_3_O_4_, FeS_2_, Sb_2_S_3_, and P, have been explored owing to their high capacity and low cost.^1^, ^2^, ^3^, ^4^, ^5^, ^6^, ^7^ Unlike intercalation reaction, conversion and alloying reactions generally involve abrupt crystallographic changes and huge volume expansion rate over 100% leading to capacity degradation by active materials destruction.^3^, ^8^, ^9^, ^10^ Although carbon and polymer materials have been used as coating layer or composite mixtures for the active materials^1^, ^3^, ^4^, ^5^, ^8^, ^9^, ^11^ to prevent pulverization, such modifications are somewhat costly and are not the fundamental solution.^5^, ^9^, ^12^

There have been numerous reports on conversion reaction materials that show the capacity recovery after its initial degradation,^13^, ^14^, ^15^ which is counter‐intuitive since conversion reaction typically induces pulverization, which translates directly into capacity degradation. Hence, we suspect that these materials could exhibit different crystallographic behavior upon sodiation to minimize pulverization. Hence, as a representative system, CuS is investigated for its capacity recovery behavior upon sodiation/de‐sodiation cycling from a microscopic perspective using in/ex situ transmission electron microscopy (TEM) in conjunction with electrochemical characterizations.

## 2 Result and discussion

### 2.1 Capacity Recovery of CuS Nanoplates

**Figure 1:**
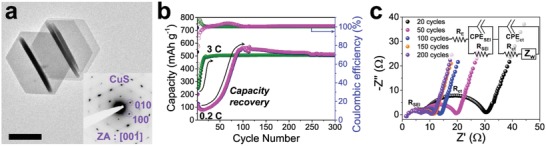
Capacity recovery of CuS nanoplates. a) Low magnification TEM image of pristine CuS nanoplates and corresponding selected area electron diffraction (SAED) pattern (scale bar, 200 nm). b) Cyclic performance of CuS nanoplates at 0.2 C and 3 C during 300 cycles. c) Nyquist plot from EIS result of CuS nanoplates during 200 cycles at 0.2 C. Semi‐circles in high frequency and high‐medium frequency regions are associated with *R*_SEI_ and *R*_ct_, respectively. *R*_E_ corresponds to ohmic resistance. CPE_SEI_ and CPE_ct_ indicate constant phase elements in SEI and charge transference.

CuS nanoplates (space group: *P*6_3_/*mmc*, **Figure** **1**a and Figure S1, Supporting Information) are charged and discharged between 0.05 and 2.6 V repeatedly at current density of 0.2 C (0.112 A g^−1^) and 3 C (1.68 A g^−1^) (Figure 1b). From electrochemical cycling, there are two observations worth noting. First, CuS shows multiple voltage plateaus (Figure S2, Supporting Information) indicating multistep phase transition via following electrochemical reactions^15^

(1) $$\begin{array}{l} \mathrm{Intercalation}:\;\mathrm{CuS}\to \mathrm{Na}{\left(\mathrm{CuS}\right)}_{4}\to {\mathrm{Na}}_{7}{\left({\mathrm{Cu}}_{6}S_{5}\right)}_{2}\\ \;\;\;\;\;\;\;\;\;\;\;\;\;\;\;\;\;\;\;\;\;\;\;\;\;\;\;\;\;\to {\mathrm{Na}}_{3}{\left(\mathrm{CuS}\right)}_{4}\;(\mathrm{to}\;\approx 0.5\;V) \end{array}$$

(2) $$\mathrm{Conversion}:\;{\mathrm{Na}}_{3}{\left(\mathrm{CuS}\right)}_{4}\to {\mathrm{Na}}_{2}S\;+\;\mathrm{Cu}\;(\mathrm{from}\;\approx 0.5\;\mathrm{to}\;0.\mathrm{05}\;V)\;$$

The intercalation process in CuS involves successive crystallographic tuning with many intermediate phases having similar crystal structures to each other.^15^ Second, the material exhibits unique capacity recovery behavior, which is quite contradictory to typical conversion reaction systems that experience severe capacity degradation upon cycling.^5^, ^9^, ^12^ At current density of 0.2 C, CuS nanoplates experience a severe capacity drop to ≈80 mAh g^−1^ after the first 13 cycles. However, the capacity gradually recovers up to ≈570 mAh g^−1^, close to its theoretical capacity, over the following 90 cycles. Interestingly, at higher current density of (i.e., 3 C), the capacity recovers just within 20 cycles after its initial drop to ≈246 mAh g^−1^.

To search for the origin of the recovery capability of CuS, we first examine changes in its electrical characteristics during 200 cycles by electrochemical impedance spectroscopy (EIS) (Figure 1c). With sodiation and desodiation cycles, both charge transfer resistance (*R*_ct_) and solid‐electrolyte interphase (SEI) layer resistance (*R*_SEI_) gradually decrease. This strongly implies that the initial capacity drop and subsequent recovery are related to both the enlargement of the active surface area and the stabilization of a SEI layer.

In other words, two conditions are to be satisfied upon the electrochemical cycling: i) a gradual increase in the active surface area, inherently insufficient in pristine state, without active materials loss and ii) formation of a highly stable SEI layer. Hence, we employ TEM to examine the active surface area generation and the SEI layer formation upon cycling to scrutinize the capacity recovery mechanism.

### 2.2 Morphological Evolution of CuS Nanoplates upon Cycling

**Figure 2:**
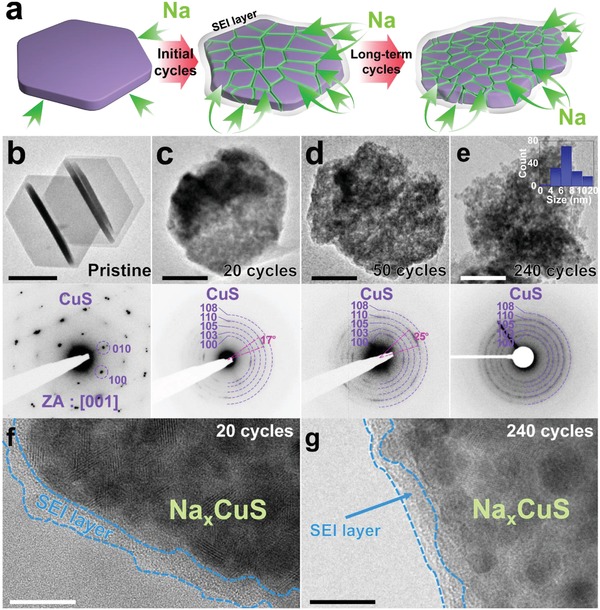
Ex situ observation of CuS nanoplates disintegration. a) Schematic model demonstrating the disintegration in CuS nanoplates. Low magnification TEM images and corresponding SAED patterns of b) pristine CuS (scale bar, 200 nm) and desodiated CuS nanoplates (scale bar, 100 nm) after c) 20 cycles, d) 50 cycles and e) 240 cycles at 0.2 C. Inset graph in (d) shows size distribution of CuS nanograins. TEM images of the SEI layers on the surface of Na*_x_*CuS after f) 20 cycles (scale bar, 20 nm) and g) 240 cycles (scale bar, 10 nm).

In order to understand the capacity recovery behavior of CuS, ex situ TEM observation of cycled CuS nanoplates is conducted as presented in **Figure** **2**. A schematic model in Figure 2a describes the steady, homogenous disintegration of CuS into small grainy parts by the sodium insertion‐induced stress, yet retaining its hexagonal plate morphology upon cycling. (Figure 2b–e). With the split of a single CuS crystal into small grains with sizes of 1–20 nm, diffraction spots ultimately turn into ring patterns (Figure S3, Supporting Information). This already suggests an increase in the exposed active surface area of CuS for facile sodium insertion/extraction and well reflects the decrease of *R*_ct_, which results in the capacity recovery. Coulombic efficiency over 100% both at 0.2 C and 3 C during the capacity recovery implies increasing extraction of sodium, which is originally confined within Na*_x_*CuS before its structural disintegration.

The disintegrated CuS well retains the original morphology after 20, 50, and 240 cycles with stable SEI layers (Figure 2f,g), composed of Na_2_CO_3_, Na_2_O, NaF, NaP*_x_*F*_y_*, and RCOONa (Figures S4 and S5, Supporting Information).^16^, ^17^, ^18^, ^19^, ^20^ Even after long‐term cycles, the SEI layer holds its morphology well to protect Na*_x_*CuS from pulverization.

**Figure 3:**
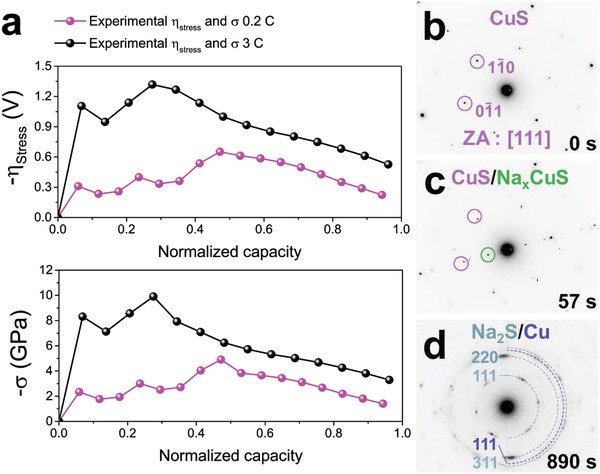
Stress‐induced overpotential and corresponding stress profiles of CuS nanoplates in conjunction with in situ diffraction pattern changes during the first cycle. a) The stress‐induced overpotential and the stress profiles from the experiment during the first cycle at 0.2 C and 3 C. In situ observation of diffraction pattern changes from b) a single pristine CuS nanoplate to c) CuS/Na*_x_*CuS and to d) fully sodiated phases (Na_2_S/Cu). CuS experiences elastic deformation upon initial Na insertion until it reaches to yield point. After touching the yield strength, plastic deformation begins with further increase of the stress. However, the intercalation phase still retains a single spot, meaning that disintegration rarely occurs in intercalation stage. Once the stress reaches to ultimate strength, it is relieved by forming Na_2_S grains through the conversion reaction, showing the diffuse diffraction spots. The yield point becomes much higher at 3 C, which originates from yield's strength variation on strain rates. The yield strength dramatically increases once it becomes larger than a critical strain rate.^36^ The stress relaxation point moves forward at 3 C due to the reaction limited intercalation reaction.^15^ Large sodium insertion into CuS induces coexistence of the intercalation and the conversion phases. Although the intercalation reaction initiates first, the conversion reaction occurs before the intercalation reaction is finished. Finally, the intercalation area caught up soon by the conversion area at high current density.^15^ As a result, stress is relieved earlier at 3 C than at 0.2 C. η_stress_ and σ corresponds to stress‐induced overpotential and stress, respectively.

Indeed, the disintegration of CuS nanoplates is caused by sodium insertion‐induced stress. Hence, in order to investigate the relationship between the electrochemically driven stress and disintegration of CuS, qualitative stress profile is obtained based on modified Butler–Volmer equation including a stress term (**Figure** **3**a)^21^, ^22^

(3) $$\eta \;=E_{V}\;-\;E_{\mathrm{eq}}=\frac{2RT}{F}\;\mathrm{arcsinh}\left(\frac{i}{2i_{0}}\right)\;\;+\frac{\sigma_{h}\Omega }{F}$$

Here,*** **η* is total overpotential, which is obtained utilizing a galvanostatic intermittent titration technique (GITT) (Figure S6, Supporting Information). Second term in the right‐hand side indicates stress‐induced overpotential. *F* corresponds to Faraday constant, equivalent of 96 485.34 C mol^−1^. *R* and *T* are ideal gas constant and absolute temperature. *E*_V_ and *E*_eq_ represent the operating and equilibrium voltages, respectively. α, σ_h_, and Ω are charge transfer coefficient, hydrostatic pressure, and partial molar volume of CuS, respectively. Finally, *i*_0_ is the exchange current density. From the measured stress profile in the intercalation regime confirmed by diffraction analysis (i.e., clear intercalation‐induced spots marked by green circles in Figure 3c and Figure S7, Supporting Information), Na*_x_*CuS well accommodates sodium insertion‐induced stress during the intercalation reaction with relative low volume expansion rate of 48%. However, during the conversion reaction, higher volume expansion rate of 98% induces the disintegration of Na*_x_*CuS nanoplates into smaller parts for effective stress relaxation (Figure 3d). Volume expansion rate is theoretically obtained from molar volume changes among CuS, 0.25Na_3_(CuS)_4_, and Na_2_S + Cu. Despite large volume change, Na*_x_*CuS nanoplates still retain their original morphologies without any crack formation. Considering that the stress is strictly induced by the overpotential, the capacity recovery is facilitated at higher current density due to high stress imposed during conversion reaction.

Based on overall structural and electrochemical assessments, Na*_x_*CuS nanoplates are indeed pulverization‐tolerant for long‐term sodium storage despite their large volume expansion in conversion reaction, which sets them distinctively apart from other electrode materials with fast pulverization.^5^, ^8^, ^9^, ^10^, ^12^

### 2.3 High‐Resolution TEM Observation of Defects within Na*_x_*CuS Nanoplates

The capacity recovery and pulverization tolerance during sodiation of CuS nanoplates are closely related to their unique crystal structural evolution. Hence, to examine it in greater detail, we conduct in/ex situ HR‐TEM observation on CuS sodiation.

**Figure 4:**
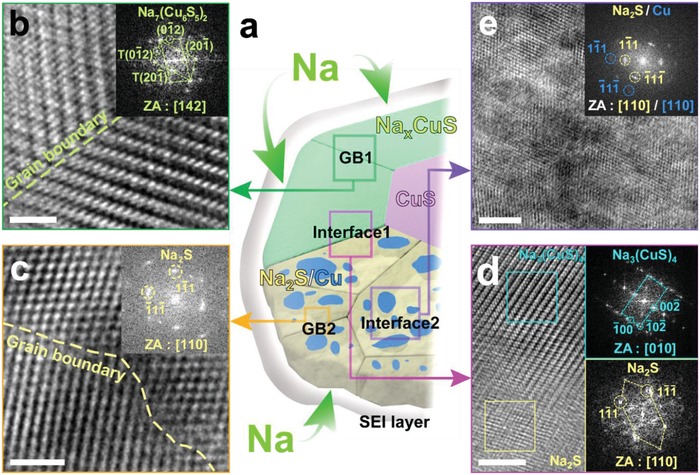
HR‐TEM observation of grain boundaries and phase interfaces in Na*_x_*CuS. a) Schematic model demonstrating grain boundaries and phase interfaces formations in Na*_x_*CuS phases. HR‐TEM images of grain boundaries formed by b) different Na inserting orientation (scale bar, 2 nm) and c) stress relaxation during the conversion reaction (scale bar, 2 nm). HR‐TEM images of phase interfaces between d) the intercalation (Na_3_(CuS)_4_) and the conversion (Na_2_S) phases (scale bar, 5 nm), and between e) Na_2_S and Cu (scale bar, 5 nm). Diffuse FFT spots of Na_2_S and Cu in (c,d) indicate that a number of Na_2_S and Cu grains are mis‐orientated from one another. GB1 and GB2 in the schematic model correspond to grain boundaries formed in the intercalation and the conversion reaction, respectively. The HR‐TEM images are Wein‐filtered.

The single crystal CuS forms grain boundaries (GBs) and semi‐coherent phase interfaces among the intercalation and the conversion phases as shown in a schematic model in **Figure** **4**a. GBs provide additional paths connected to the active sodium ion diffusion channels of Na*_x_*CuS,^23^ while semi‐coherent phase interfaces act as mechanical pillars that prevent the structural pulverization.^24^, ^25^, ^26^

GBs can be generated i) between two differently oriented intercalation phase and ii) upon mechanical stress relaxation during the conversion reaction. During the intercalation reaction, sodium is inserted through the active channels along {001} planes of CuS to initiate two intercalation reaction fronts along different orientations (Figure S7, Supporting Information). The two reaction fronts overlap with each other to form twin boundaries among Na*_x_*CuS grains (Figure 4b and Figure S8b, Supporting Information). Ultimately, after the conversion reaction, Na_2_S grains with different orientation can be formed (Figure S9, Supporting Information). In the conversion reaction, GBs are formed by stress relaxation (Figure 4c). Indeed, diffuse electron diffractions and fast‐Fourier transform (FFT) spots from Na_2_S and Cu imply that a number of Na_2_S and Cu grains are slightly mis‐orientated from one another over whole particle (Figure 4c and Figure S10, Supporting Information).

The two crucial semi‐coherent phase interfaces with the conversion reaction are phase interfaces i) between intercalation and conversion phases (Na_3_(CuS)_4_/Na_2_S, Figure 4d), and ii) between conversion phases (Na_2_S/Cu, Figure 4e). The former is generated by an encounter of the two planes; $\left\{\bar{1}0\bar{2}\right\}$ plane of Na_3_(CuS)_4_ and $\left\{1\bar{1}\bar{1}\right\}$ plane of Na_2_S with the periodicity difference of only about 2.5% (Figure S8d, Supporting Information). Therefore, in the interface, sodium is inserted into the centers of CuS*_x_* columns, replacing the copper atoms in the columns to form Na_2_S. The latter is formed by extracted copper in the conversion reaction by having it aligned coherently with a Na_2_S matrix based on their same face centered cubic (FCC) structure (Figure 4e and Figure S8c, Supporting Information). As a result, both Na_2_S and Cu have {111} planes aligned along [110] direction. The latter coherency between Na_2_S and uniformly distributed Cu contributes to cyclic stability by avoiding demerits induced by copper agglomeration. Copper agglomeration inside nanoplates can aggravate the pulverization of Na_2_S matrix while copper dendrite growth outside the nanoplates would engender a contact loss among the active material, conductive carbon, and binder. For instance of the latter case, lithium storage in CuS forms copper dendrite outside Li_2_S matrix (Figure S11, Supporting Information), which is driven by high copper mobility, and almost same sulfur lattices and molar volumes between Cu_2−_ *_x_*S and Li_2_S.^27^ As a result, copper agglomeration hampers reversible delithiation back to CuS again and ultimately induces gradual decrease of lithium storing capacity (Figure S12, Supporting Information).

The semi‐coherent phase interfaces induce stress relaxation by forming grains and grain boundaries rather than random pulverization. As a result, CuS nanoplates well retain the recovered capacity and the original morphology even after full disintegration. The abnormal phenomenon originates from the semi‐coherent conversion reaction interface. For generation of the interface, synergistic effect between large sodium and high copper diffusivity facilitates multiple phase transitions. Copper ionic diffusivity in CuS is much higher (${\tilde{D}}_{\mathrm{Cu}}=\;2.5\;\times {\mathrm{10}}^{\mathrm{–12}}\;{\mathrm{cm}}^{2}\;s^{\mathrm{–1}}$) than other cations in metal sulfides like FeS_2_ (${\tilde{D}}_{\mathrm{Fe}}=\;\approx {\mathrm{10}}^{\mathrm{–17}}\;\;{\mathrm{cm}}^{2}\;s^{\mathrm{–1}}$ at 100 °C).^28^, ^29^ Large sodium cannot replace copper atoms unlike lithiation case in CuS due to high formation energy, which engenders formation of Na‐Cu‐S ternary system to generate Na_3_(CuS)_4_/Na_2_S semi‐coherent boundary (Figure S13, Supporting Information).

### 2.4 Perspective on Practical Application of CuS

**Figure 5:**
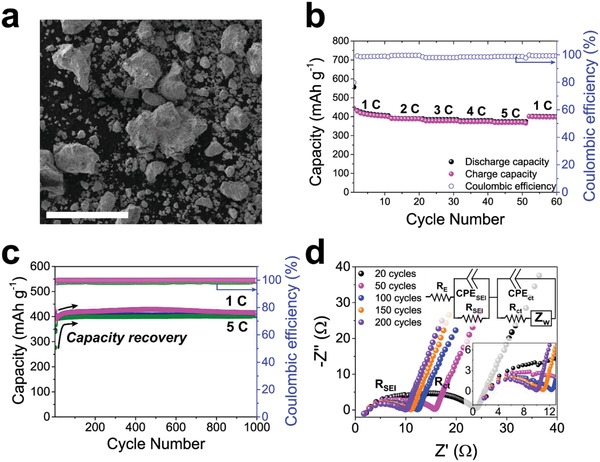
Electrochemical performance of bare bulk CuS. a) SEM image of bulk CuS (scale bar, 100 µm), and its b) C‐rate capability from 1 C to 5 C and c) cyclic performance at 1 C and 5 C during 1000 cycles. d) EIS result obtained from bulk CuS within the frequency range between 1000 kHz and 0.1 Hz at amplitude of 10 mV. Inset graph in (d) is magnified high frequency region.

For practical application of CuS electrodes for sodium ion batteries, it is important to understand their compatibility in a bulk form since size and morphological tuning likely involve high‐cost and complex synthesis. In such a context, we successfully demonstrate that bulk CuS also (**Figure** **5**a and Figure S14a, Supporting Information) exhibits high sodium storage performance even without size and shape optimization.

Bulk CuS with hundreds of micrometer in size exhibits high capacity and exceptional stability at various current densities ranging from 1 C to 5 C (Figure 5b,c). In addition, bulk CuS presents long‐term cyclic stability by retaining the specific capacity of 415 and 406 mAh g^−1^ after 1000 cycles at 1 C and 5 C, respectively. Cyclic stability is maintained even after 2100 cycles with a capacity retention of ≈93% after the second cycle (Figure S15, Supporting Information). Bulk CuS also shows the capacity recovery behavior, electrical property changes, and discharge plateaus similar to those of CuS nanoplates (Figure S14b, Supporting Information). At 1 C, bulk CuS initially experiences a slight capacity drop to 392 mAh g^−1^ during the initial 8 cycles. However, the capacity is recovered up to 429 mAh g^−1^ over the next 500 cycles. In the case of higher current density of 5 C, the capacity is recovered faster for 100 cycles after a more severe capacity drop to 276 mAh g^−1^.

Comparing with CuS nanoplates, the initial capacity drops are alleviated in bulk particles due to the relative larger active surface area than that of nanoplates of which the surface area is mainly composed of electrochemically inactive {001} planes (Figure S7, Supporting Information). Furthermore, once the initial discharge occurs at relative low current density (i.e., 1 C), a capacity drop at high current density (i.e., 5 C) does not occur (Figure 5b,c). In other words, initial slow discharge enables stable operation at higher current density, which clearly suggests the practical viability of bulk CuS for real battery application.

On the other hand, with the initial discharge at much higher current density of 10 C, enormous overpotential hampers sodium insertion resulting in very low first discharge capacity of ≈46 mAh g^−1^. As a result, bulk CuS does not undergo proper conversion reaction, lowering the capacity recovery rate (Figure S16, Supporting Information). Therefore, slow initial discharge should be preceded before high current density operation. Nevertheless, based on the similar electrochemical behaviors of CuS in both bulk and nanoplate forms, it is safe to conclude that CuS inherently possesses capacity recovery and pulverization‐tolerant characteristics for sodium storage regardless of its size.

### 2.5 Comparison with Lithium Ion Batteries

The results presented above offer the possibility of using CuS as an anode of SIBs. To understand the feasibility for its practical application for energy storage, we compare CuS with Li_4_Ti_5_O_12_ (LTO), which is conventional LIB anode for ESS due to its superior cyclic stability. **Table** **1** presents the average charge voltage and raw materials cost per capacity for CuS and LTO. CuS shows 26% lower raw materials cost per capacity than LTO.

**Table 1:** Cost comparison between CuS and LTO anodes. Raw materials prices are obtained from Korea Mineral Resource Information Service, Shanghai Metal Market and United State Geological Survey

Electrode materials/Elements	Theoretical capacity [mAh g^−1^]	Average charge voltage [V]	Raw materials	Raw materials prices [usd ton^−1^]	Required raw materials cost [usd ton^−1^]	Raw materials cost per capacity [10^−6^ usd mAh^−1^]
Li_4_Ti_5_O_12_	175	1.6			4008	22.902
Li			Li_2_CO_3_	9675^a)^		
Ti			TiO_2_	850^c)^		
CuS	560	1.55			3437	6.138
Cu			CuSO_4_·5H_2_O	2137.5^b)^		
S			S	60^c)^		

^a)^ Korea Mineral Resource Information Service (www.kores.net)

^b)^ Shanghai Metal Market (www.smm.cn)

^c)^ United State Geological Survey on 14 April 2019.

LTO is generally combined with Li(Ni*_x_*Co*_y_*Mn*_z_*)O_2_ (NCM) (or Li(Ni*_x_*Co*_y_*Al*_z_*)O_2_ (NCA), *x* + *y* + *z* = 1) having an average discharge voltage of ≈3.8 V (vs Li/Li^+^) for full cell configuration. Therefore, if CuS is combined with a suitable cathode material having a similar average discharge voltage, we can expect that such a full cell competes with the existing LTO‐NCM (or LTO‐NCA) battery for ESS from the low cost perspective. Among various cathode candidates, Na_3_V_2_(PO_4_)_2_F_3_ (NVPF) and Na_2_Fe_2_(SO_3_)_4_ (NFS) are considered as viable options owing to their high discharge potential of ≈3.8 V (Na/Na^+^).^30^, ^31^ To investigate the cost competitiveness of a CuS anode based full cell, we does not only present raw materials costs for NMC, NVPF, and NFS (**Table** **2**), but also compare CuS anode‐based full cell and the LTO‐NMC cell in **Table** **3**. Although NVPF has been intensively studied due to its high redox potential and high capacity, a recent surge in the price of vanadium directly lead to an increase in the cost of NVPF making it less viable than NFS with earth‐abundant iron. Raw materials cost for CuS‐NFS is only ≈10% of LTO‐NCM while its gravimetric capacity is only ≈9% lower than that of LTO‐NCM.

**Table 2:** Cost comparison among NMC, NVPF and NFS cathodes. Raw materials prices are obtained from Korea Mineral Resource Information Service, Shanghai Metal Market, United State Geological Survey, Vanadium prices, and Alibaba. For cathode materials, practical capacity is used due to large gap between theoretical and practical values

Electrode materials/Elements	Practical capacity [mAh g^−1^]	Average discharge voltage [V]	Raw materials	Raw materials prices [usd ton^−1^]		Required total raw materials cost [usd ton^−1^]	Raw materials cost per capacity[10^−6^ usd mAh^−1^]
NCM (LiNi_0.8_Co_0.1_Mn_0.1_O_2_)	205	3.8				14115	68.85
Li			Li_2_CO_3_	9675^a)^			
Ni			NiSO_4_·6H_2_O	3975^b)^			
Co			CoSO_4_ 7H_2_O	7800^b)^			
Mn			MnSO_4_	1020^b)^			
NVPF (Na_3_V_2_(PO_4_)_2_F_3_)	120	3.8				13563	113.03
Na/F			NaF	800c), e)			
V			V_2_O_5_	29778^d)^			
PO_4_			NH_4_H_2_PO_4_	650^e)^			
NFS (Na_2_Fe_2_(SO_3_)_4_)	100	3.8				140	1.4
Na			Na_2_SO_4_	150c), e)			
Fe			FeSO_4_·7H_2_O	150^e)^			

^a)^ Korea Mineral Resource Information Service (www.kores.net)

^b)^ Shanghai Metal Market (www.smm.cn)

^c)^ United State Geological Survey

^d)^ Vanadium Prices (www.vanadiumprice.com)

^e)^ Alibaba on 14 April 2019.

**Table 3:** Cost comparison among LTO‐NCM, CuS‐NVPF and CuS‐NFS full cell configuration

Anode/cathode combination	Required anode mass [g]	Required cathode mass [g]	Gravimetric capacity [mAh g^−1^]	Required raw materials cost [usd]
LTO‐NCM	18.9	14.6	89.55	0.293
CuS‐NFS	30	5.9	83.57	0.024
CuS‐NVPF	23.4	5.9	102.39	0.338

1) Full cell capacity = 3000 mAh

2) Anode and cathode capacity ratio = 1.1

3) Gravimetric capacity is based on total electrodes mass.

## 3 Conclusion

We reveal the pulverization tolerant, capacity recovery mechanisms in sodiation of CuS nanoplates. Formation of stable grain boundaries and phase interfaces in Na*_x_*CuS contributes to its capacity recovery and pulverization tolerance. To our great surprise, the mechanism above is universal as the bulk CuS also exhibits similar electrochemical performance to that of nanoplates. Based on its comparison with counterparts in LIBs, it can be considered as a viable anode candidate for sodium ion based ESS.

Our findings suggest that the crystallographic relationships among sodium‐insertion phases hold an ultimate key for mechanically robust cycling of high‐performance conversion reaction materials.

## 4 Experimental Section

*CuS Bulk and Nanoplate Preparation*: Bulk CuS was purchased from Sigma‐Aldrich. For CuS nanoplates synthesis, solvothermal method is utilized as following.^15^, ^32^ Transparent microemulsion was obtained by stirring and sonicating a solution containing 2.5 g CTAB (in hexane), 8.5 mL *n*‐pentanol, and 1.3 mL water containing 0.057 g copper nitrate trihydrate. 0.8 mL carbon disulfide was added before the microemulsion was poured into a 100 mL Teflon‐sealed autoclave. The autoclave was treated for 15 h at 170 °C in an electric oven. After natural cooling, the black precipitation was obtained. The precipitation was dried in a vacuum oven at 60 °C for 12 h after washing with acetone and ethanol.

*Materials Characterization*: Transmission electron microscopies (JEM 3010, JEM 2100F, ARM 200, JEOL) were employed to confirm a morphology and crystal structure of CuS nanoplates. X‐ray photoelectron spectroscopy (XPS, K‐Alpha+, Thermofisher scientific) was used for SEI layer composition analysis. Scanning electron microscopy (FEI, Nova 230) was employed to confirm morphology of purchased bulk CuS (Sigma‐Aldrich, 100mesh).

*In Situ TEM Sample Preparation and Characterization*: TEM sample was prepared by dropping NaF (or LiF) particles and CuS nanoplates on a graphene coated holey carbon Au grid (300 mesh, SPI), which was prepared by direct transfer method.^33^, ^34^ NaF (or LiF) was used to generate metallic Na (or Li) from NaF (or LiF). Na (or Li) metal, generated by electron beam, directly reacted with active materials. TEMs (JEM‐2100F, JEM‐3010, JEOL) equipped with charge coupled devices (CCD) camera (Orius SC1000, US1000, Gatan) were used for observation of disintegrated Na*_x_*CuS particles and interface between the intercalation and conversion phases at the accelerating voltage of 200 kV.

*Electrochemical Cell Test and Ex Situ Characterization*: To fabricate the working electrode, slurry containing active materials, carbon black (acetylene black, Alfa Aesar), and polyvinylidene fluoride (PVDF, Sigma Aldrich) were prepared with 1‐methyl‐2‐pyrrolidone. The ratio among active materials, carbon black, and PVDF was 8:1:1. The slurry was coated on a Cu foil, and the foil was dried for 12 h in a vacuum oven. Electrolyte was prepared by dissolving NaPF_6_ into diglyme and heating at 80 °C for 48 h inside a glove box under Ar atmosphere.^35^ A pure Na foil (Sigma Aldrich) and a glass fiber (GF/F, Whatman) were used as a counter electrode and a separator, respectively. 2032 type coin cell and swagelok cell (ECC‐STD, EL‐CELL) were assembled inside a glove box under Ar atmosphere. Galvanostatic cell test was performed using a battery cycling system (WBCS 3000L, Wonatech). EIS was performed using a potentiostat (PARSTAT MC 1000, Princeton Applied Research). All electrochemical tests were performed at room temperature. For ex situ experiments, a coin cell was disassembled after number of cycles. For TEM analysis, an active material was thoroughly washed via active sonication for 3 h in diglyme and dispersed onto a grid for TEM examination. For SEI layer composition analysis, discharged electrode was washed in diglyme before XPS characterization.

*Stress Profile Calculation*: Stress‐induced overpotential profile in Figure 3 is obtained using modified Butler–Volmer equation including a stress effect^21^

(4) $$i\;=i_{0}\;\left\{\exp\left[\left(1-\alpha \right)\frac{F\left(E_{V}-E_{\mathrm{eq}}\right)-\sigma_{h}\Omega }{RT}\right]-\exp\left[-\alpha \frac{F\left(E_{V}-E_{\mathrm{eq}}\right)-\sigma_{h}\Omega }{RT}\right]\right\}$$

Here, *F* corresponds to faraday constant, 96 485.34 C mol^−1^. *R* and *T* are ideal gas constant and absolute temperature. *E*_V_ and *E*_eq_ present operating and equilibrium voltages, respectively. α, σ_h_, and Ω are charge transfer coefficient, hydrostatic pressure, and partial molar volume of CuS. *i*_0_, exchange current, is expressed as

(5) $$i_{0}=\;Fk_{0}c_{{\mathrm{Na}}^{+}}^{1-\alpha }{\left(c_{\max}-c_{\mathrm{surf}}\right)}^{1-\alpha }c_{\mathrm{sulf}}^{\alpha }\;$$

*c*_max_, *c*_surf_, and $c_{{\mathrm{Na}}^{+}}^{1-\alpha }$ correspond to maximum sodium concentration in the active material, sodium concentration at the surface of the active material and in the electrolyte. Based on the equation, assuming α  =  0.5, total overpotential is arranged like following

(6) $$\eta \;=E_{V}\;-\;E_{\mathrm{eq}}=\frac{2RT}{F}\;\mathrm{arcsinh}\left(\frac{i}{2i_{0}}\right)+\frac{\sigma_{h}\Omega }{F}$$

To acquire the stress‐induced overpotential (η_stress_) profile, first term in right side, current‐effected one, is subtracted from total overpotential (η) obtained from GITT. GITT was performed by applying pulse currents corresponding 0.2 C and 3 C for 20 min and 1 min 20 s with time interval of 2 h. For the calculation, following values are used^21^, ^22^

(7) T = 298 K

(8) $$\;k_{0}=5\;\times \;10^{-12}\;\;m^{2.5}\;{\mathrm{mol}}^{-0.5}\;s^{-1}$$

(9) $$\;C_{{\mathrm{Na}}^{+}}=\;1000\;\mathrm{mol}\;m^{-3}$$

(10) $$\;C_{\max}=\;96950\;\mathrm{mol}\;m^{-3}\;$$

(11) $$\Omega_{\mathrm{intercalation}}=\;1.287\;\times 10^{-5}\;m^{3}\;{\mathrm{mol}}^{\mathrm{–1}}$$

(12) $$\;\Omega_{\mathrm{conversion}}=\;1.544\;\times 10^{-5}\;m^{3}\;{\mathrm{mol}}^{\mathrm{–1}}$$

(13) $$i\;=\;2.12\;A\;m^{\mathrm{–2}}\left(0.2\;C\right),\;\mathrm{15}.\mathrm{74}\;A\;m^{\mathrm{–2}}\left(3\;C\right)$$

To obtain partial molar volume during intercalation reaction and conversion reaction, volume expansions of 48% and 96% are used, respectively. The volume expansion rates are calculated based on molar volumes of CuS (20.09 m^3^ mol^−1^), 0.25(Na_3_(CuS)_4_) (29.78 m^3^ mol^−1^), Cu (7.09 m^3^ mol^−1^), and Na_2_S (41.96 m^3^ mol^−1^). Two partial molar volumes are used for converting an overpotential term to a stress term in Figure 3.

## Conflict of Interest

The authors declare no conflict of interest.

## Supporting information

Supplementary
