# Supplementary material for: Pulverization‐Tolerance and Capacity Recovery of Copper Sulfide for High‐Performance Sodium Storage
Source: Adv Sci (Weinh). 2019 Apr 26;6(12):1900264. doi: 10.1002/advs.201900264 (PMC6662052; doi:10.1002/advs.201900264)
Supplement: Supplementary file 1 — Supplementary [file ADVS-6-1900264-s001.pdf]

Copyright WILEY-VCH Verlag GmbH & Co. KGaA, 69469 Weinheim, Germany, 2019.

Supporting Information

for *Adv. Sci.,* DOI: 10.1002/advs.201900264

Pulverization-Tolerance and Capacity Recovery of Copper Sulfide for High-Performance Sodium Storage

*Jae Yeol Park, Sung Joo Kim, Kanghoon Yim, Kyun Seong Dae, Yonghee Lee, Khoi Phuong Dao, Ji Su Park, Han Beom Jeong, Joon Ha Chang, Hyeon Kook Seo, Chi Won Ahn, and Jong Min Yuk**

Supporting Information

Pulverization-tolerance and capacity recovery of copper sulfide for high performance sodium storage

*Jae Yeol Park, Sung Joo Kim, Kanghoon Yim, Kyun Seong Dae, Yonghee Lee, Khoi Phuong Dao, Ji Su Park, Han Beom Jeong, Joon Ha Chang, Hyeon Kook Seo, Chi Won Ahn, and Jong Min Yuk**

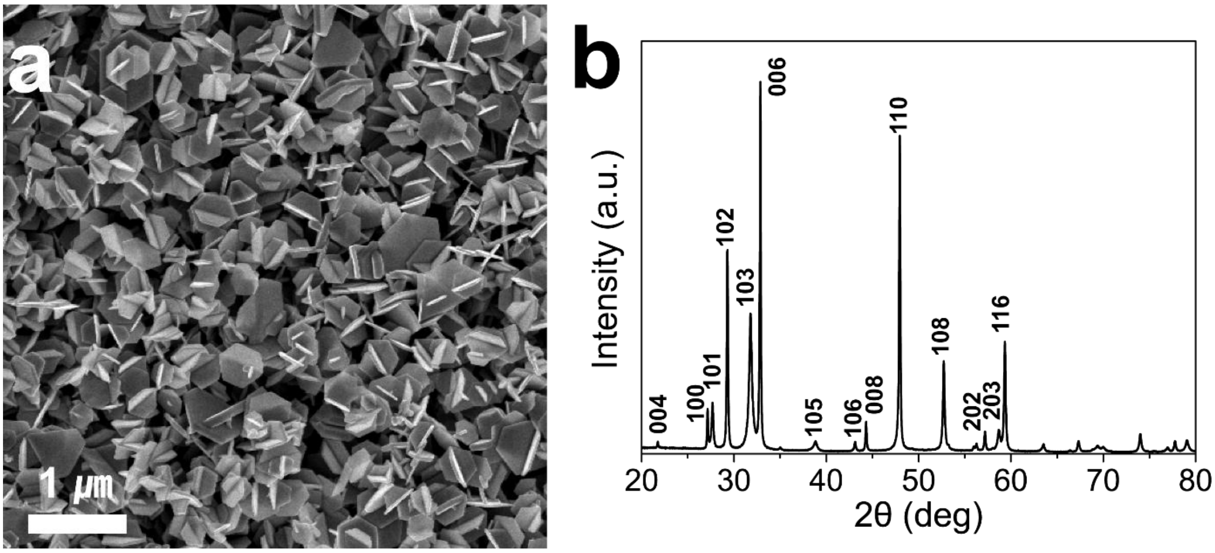

Figure S1. (a) Scanning electron microscope (SEM) image and **(b)** X-ray diffraction (XRD) pattern of CuS nanoplates.

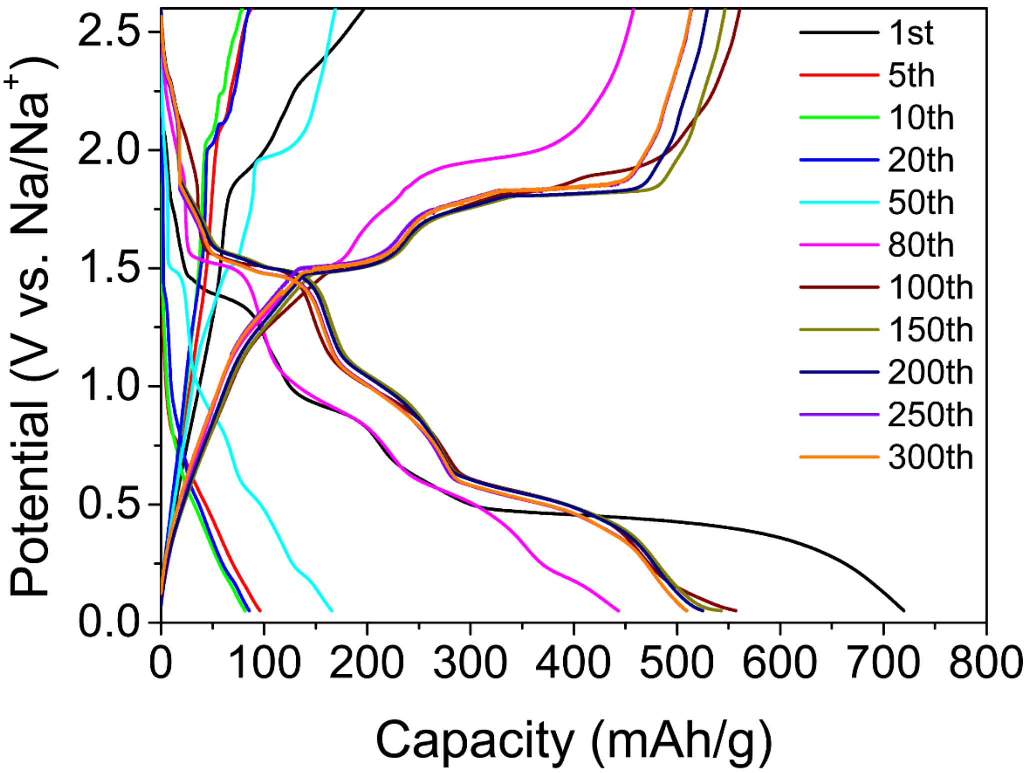

Figure S2. Charge and discharge profile of CuS nanoplates at 0.2 C.

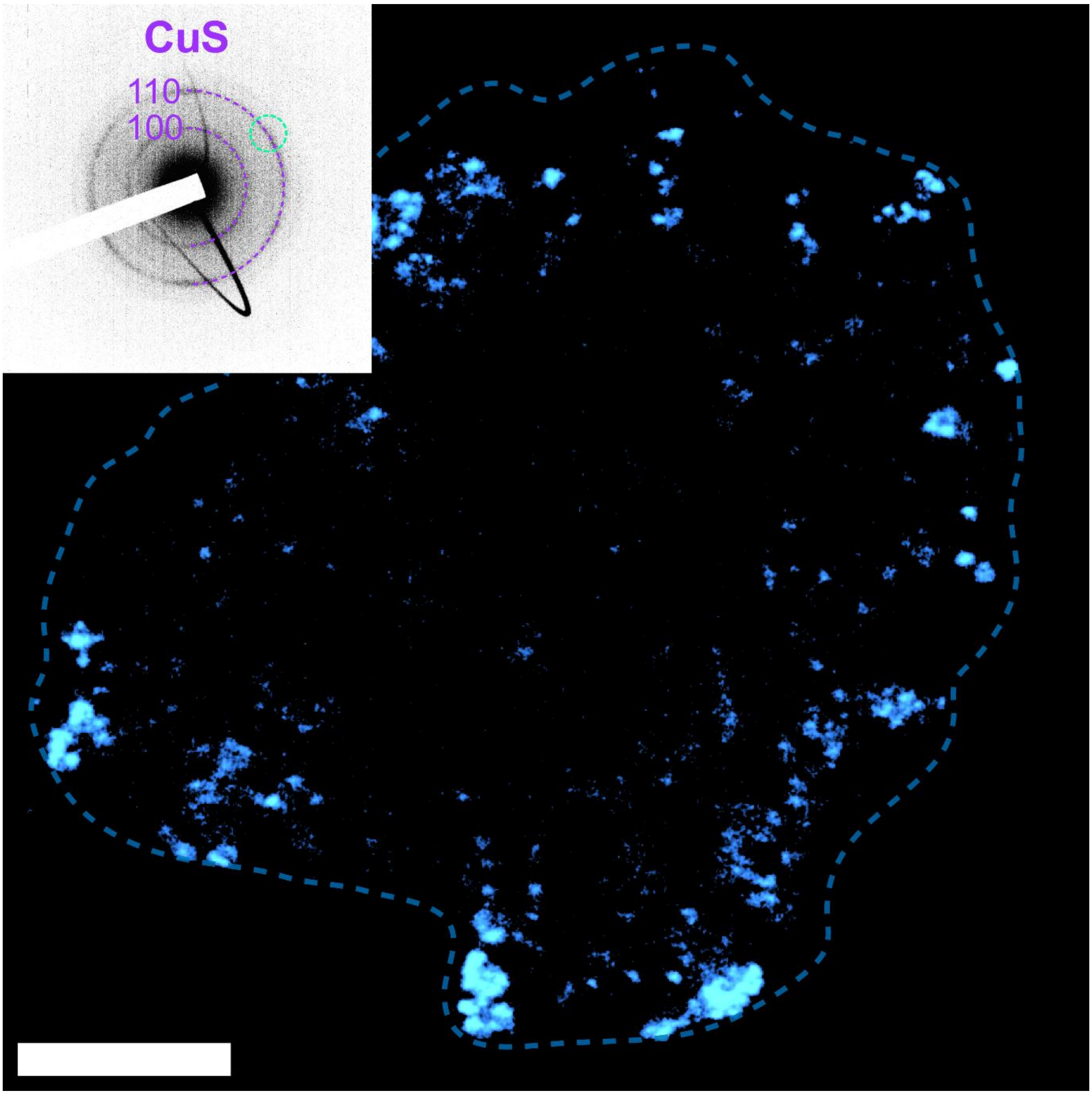

Figure S3. Nano grains in fully disintegrated CuS nanoplates. Centered dark-field (CDF) transmission electron microscope (TEM) image corresponding diffraction spots of green dotted circle (scale bar, 100 nm).

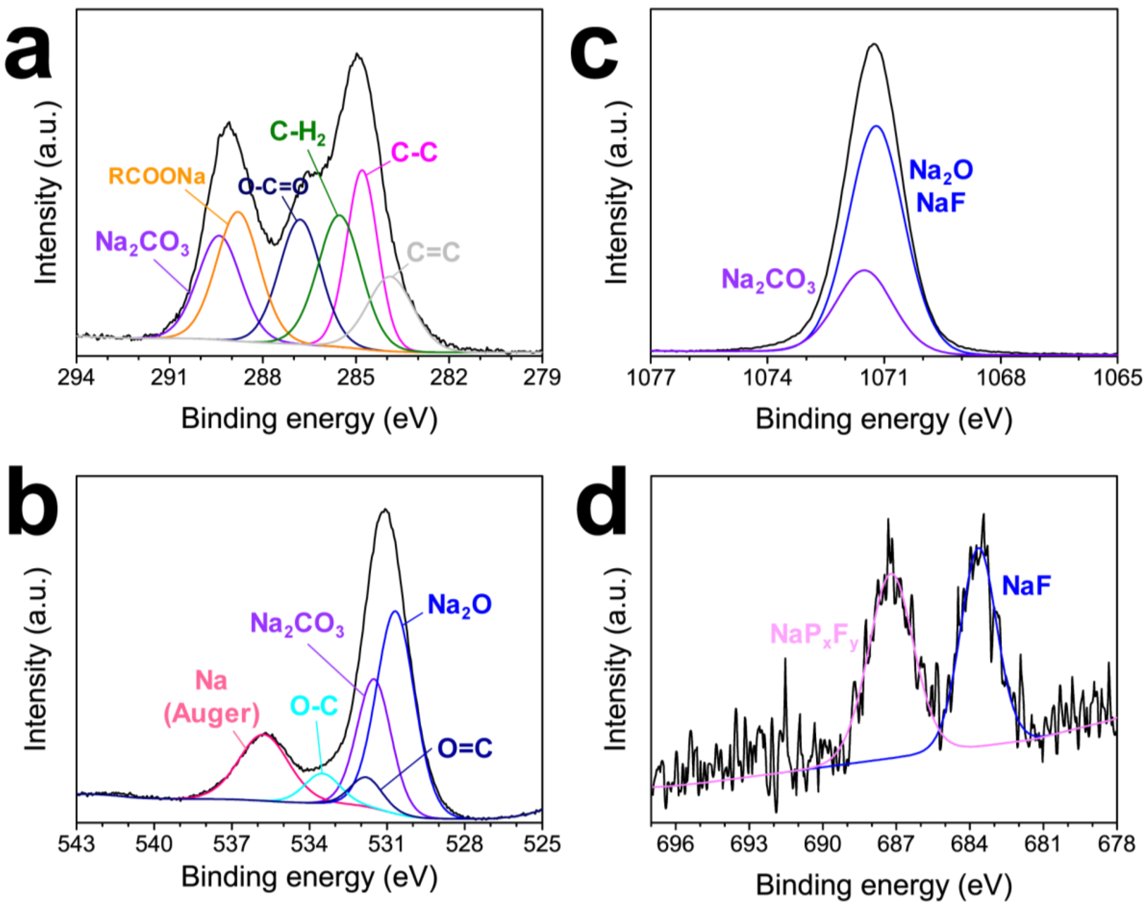

Figure S4. XPS spectra of **(a)** C1s**, (b)** O1s, **(c)** Na1s and **(d)** F1s obtained from CuS nanoplates after 20 cycles. C1s spectrum is fitted with the binding energies of 283.9 eV (C=C), 284.8 eV (C-C), 285.5 eV (C-H_2), 286.8 eV (O-C=O), 288.8 eV (RCOONa) and 289.4 eV (Na_2CO_3). O1s spectrum is fitted with the binding energies of 544.6 eV (Na_2O), 531.5 eV (Na_2CO_3), 531.8 eV (O=C), 533.5 eV (O-C) and 535.8 eV (Na Auger). Na1s spectrum is fitted with the binding energies of 1078.6 eV (Na_2O and NaF) and 1071.5 (Na_2CO_3). F1s spectrum is fitted with the binding energies of 683.6 eV (NaF) and 687.2 eV (NaP_xF_y).

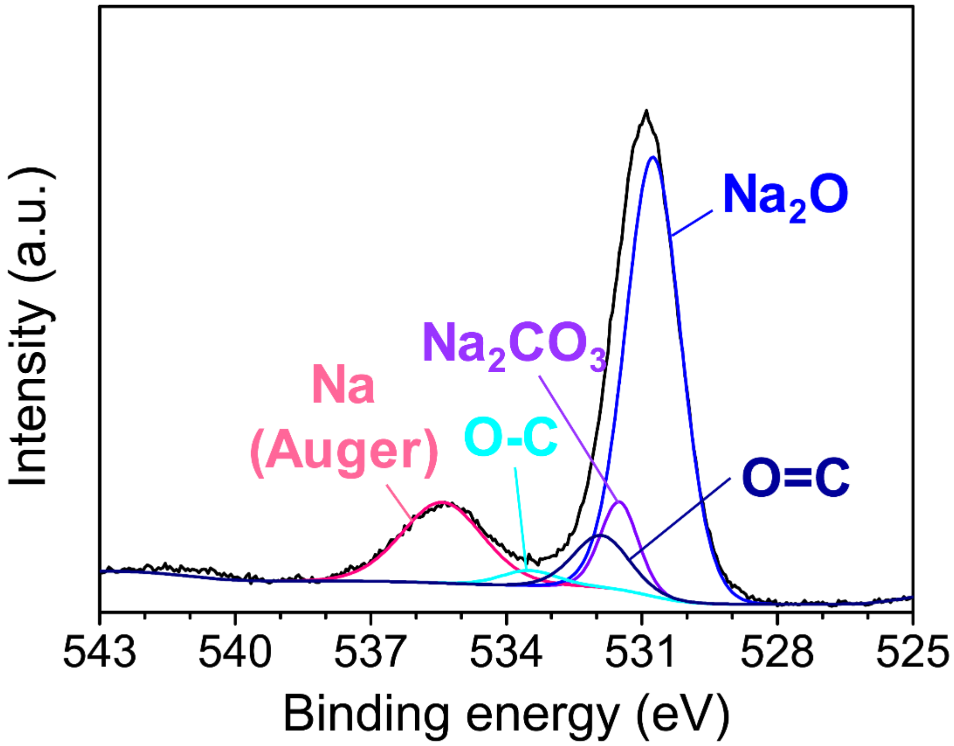

Figure S5. XPS spectrum of O1s from CuS nanoplates after the first discharge to 0.2 V. Observed sodium auger peaks in both after 20 cycles (Figure S4) and first discharge to 0.2 V indicate that the peaks are induced by solid electrolyte interphase (SEI) layer, but not other artifacts like sodium metal deposition on the electorde.

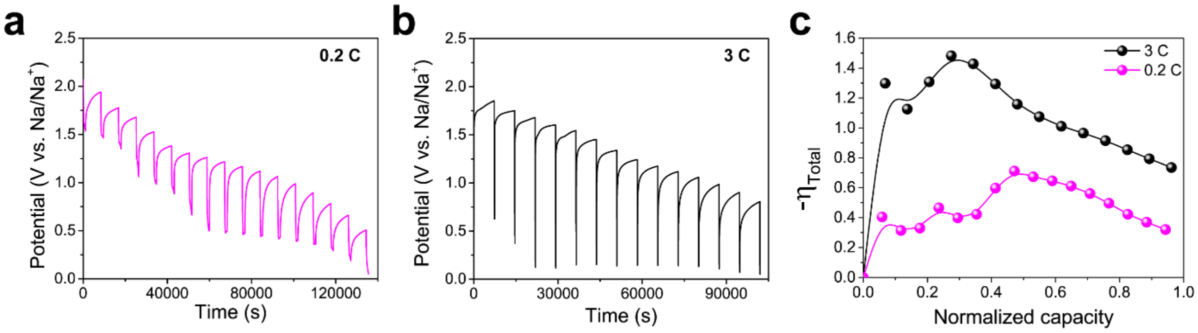

Figure S6. Total overpotential of CuS nanoplates during the first sodiation. Galvanostatic intermittent titration technique (GITT) profile for the first sodiation at **(a)** 0.2 C and **(b)** 3 C. **(c)** Total overpotential (η_{Total}) variation during the first sodiation obtained from the GITT profile.

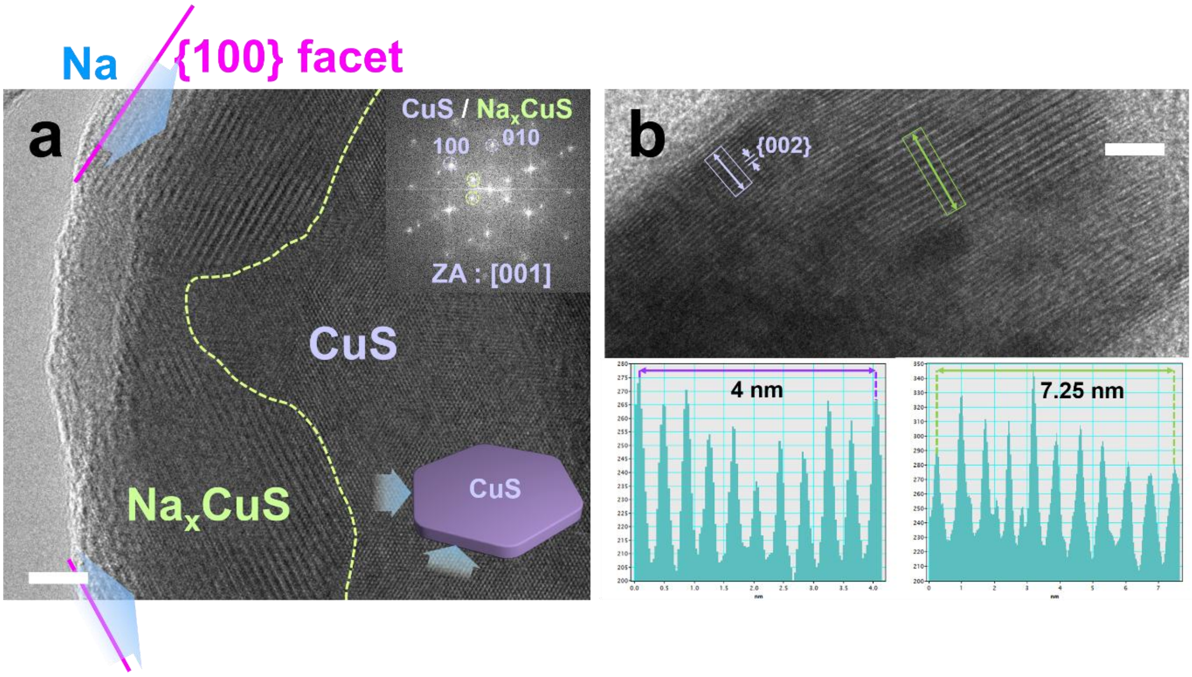

Figure S7. Sodium insertion dynamics in intercalation stage. High resolution transmission electron microscopy (HR-TEM) images (scale bars, 5 nm) demonstrating **(a)** sodium insertion through {100} planes occupying {001} plane at edge of CuS nanoplate and **(b)** lattice expansion between {001} planes. Sodium is favorably inserted into active channels composed of {001} planes, and {001} planes are electrochemically inactive. In addition based on the crystal structure of CuS ($P6_3/mmc$, hexagonal), except for {001} planes, all planes are open to the active channels and available for sodium insertion into the channels.

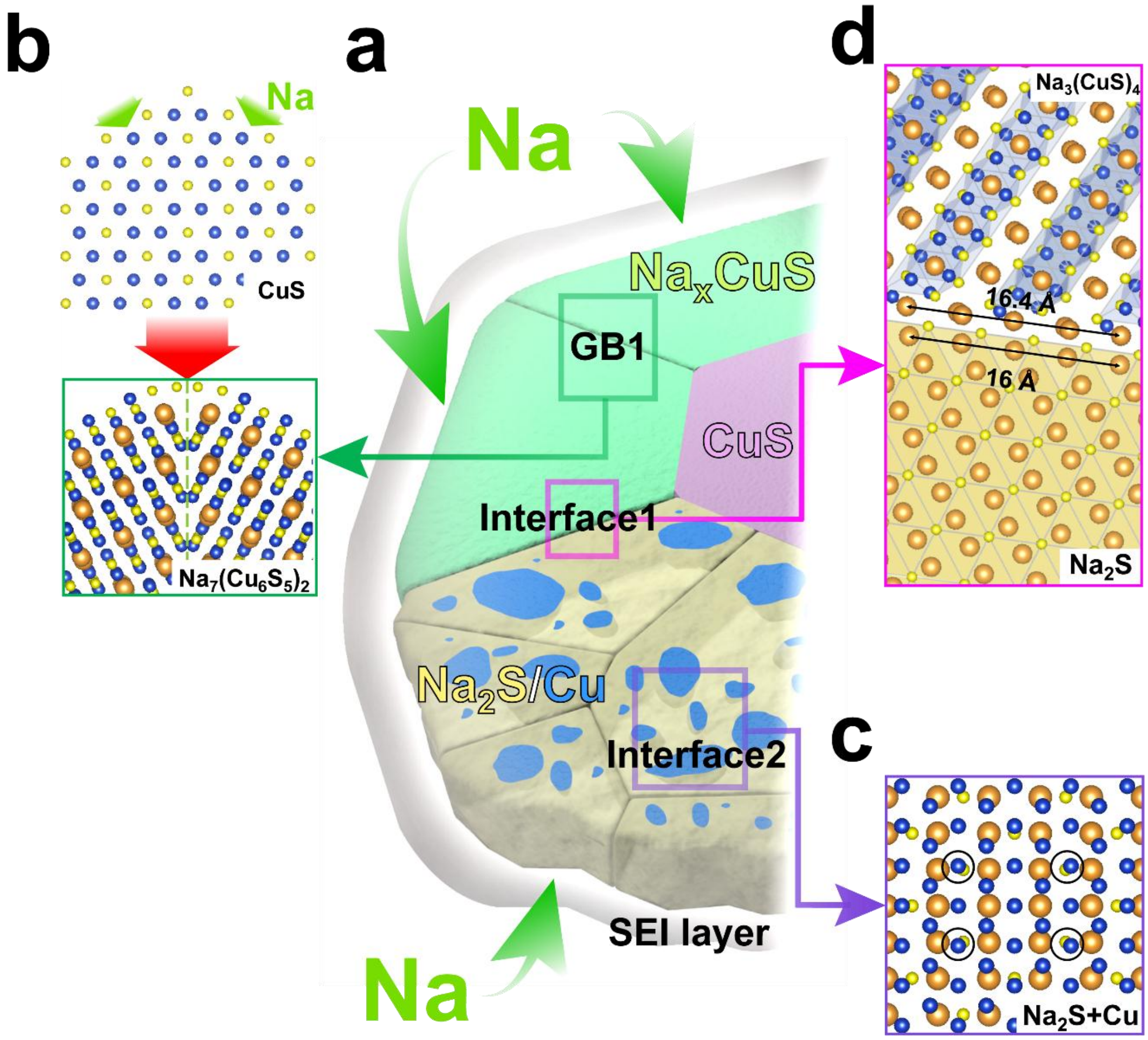

Figure S8. Atomic models of grain boundary and interfaces observed in sodiated CuS. **(a)** Schematic model demonstrating grain boundary and phase interface formations in intercalation and conversion reactions. Atomic models of **(b)** grain boundary formed by encounter of two intercalation reaction fronts, **(c)** interfaces between Na_2S and Cu, and **(d)** between intercalation and conversion phases.

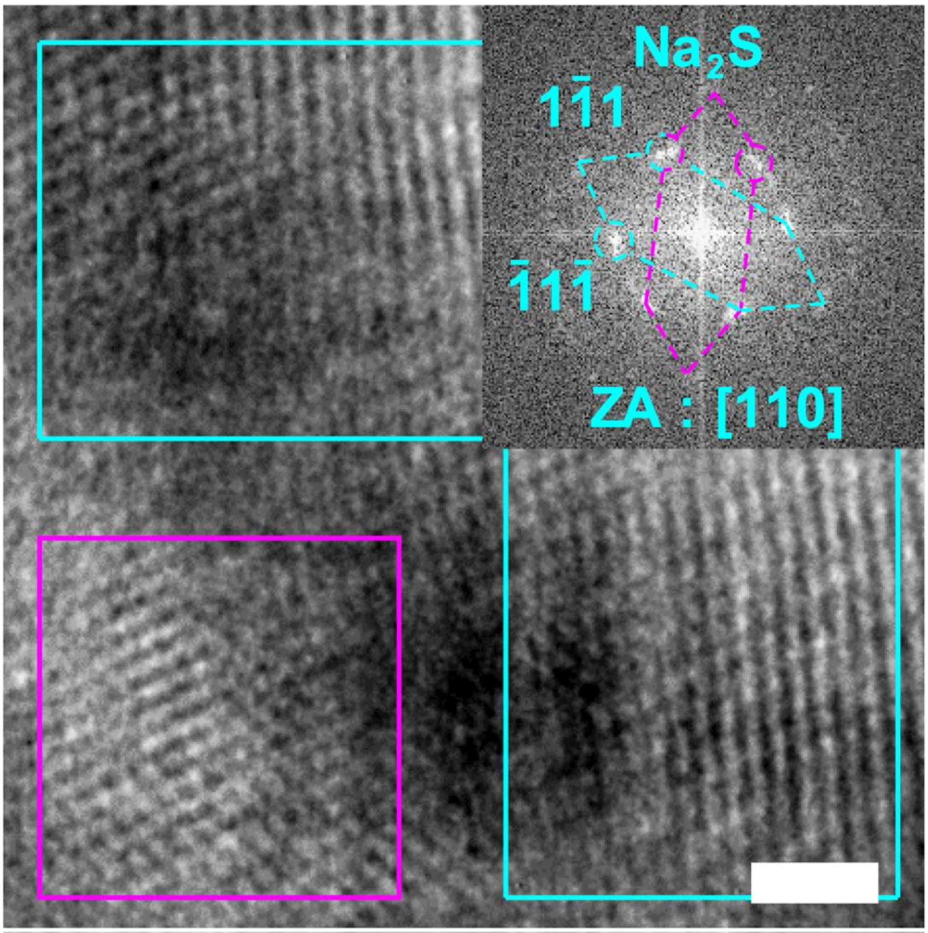

Figure S9. Wein-filtered HR-TEM images of Na_2S grains with different orientation (scale bar, 2 nm).

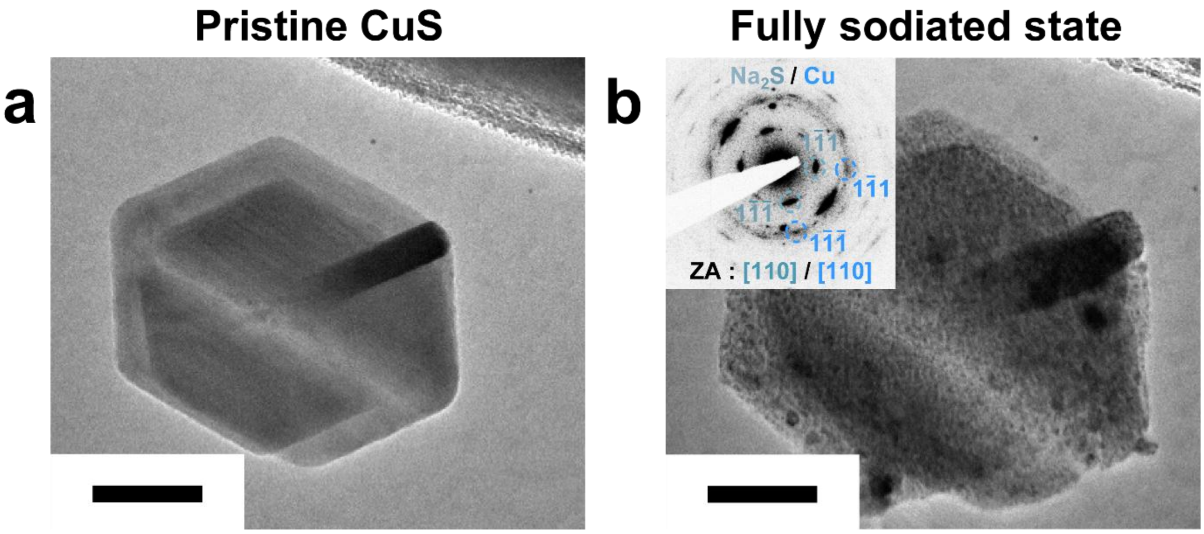

Figure S10. Sodiation of a CuS nanoplate. Low magnification TEM images (scale bars, 100 nm) of **(a)** pristine and **(b)** fully sodiated CuS nanoplate. Diffraction pattern of the fully sodiated nanoplate exhibits coherence between Na_2S and Cu over whole particle.

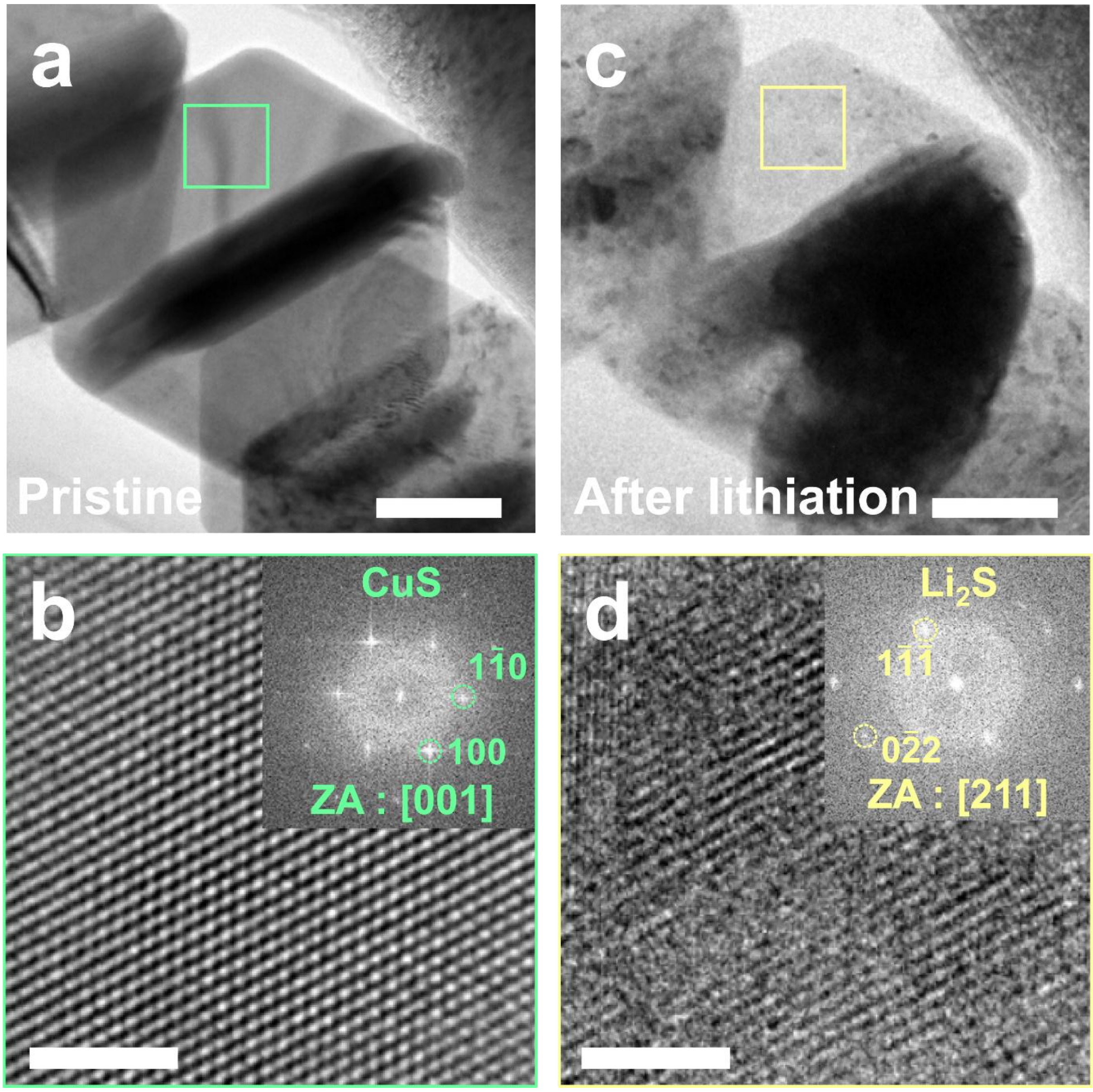

Figure S11. Copper dendrite formation in CuS lithiation. Low magnification and HR-TEM images of CuS **(a)(b)** before and **(c)(d)** after lithiation. (Scale bars in (a),(c) are 100 nm and in (b),(d) are 5 nm)

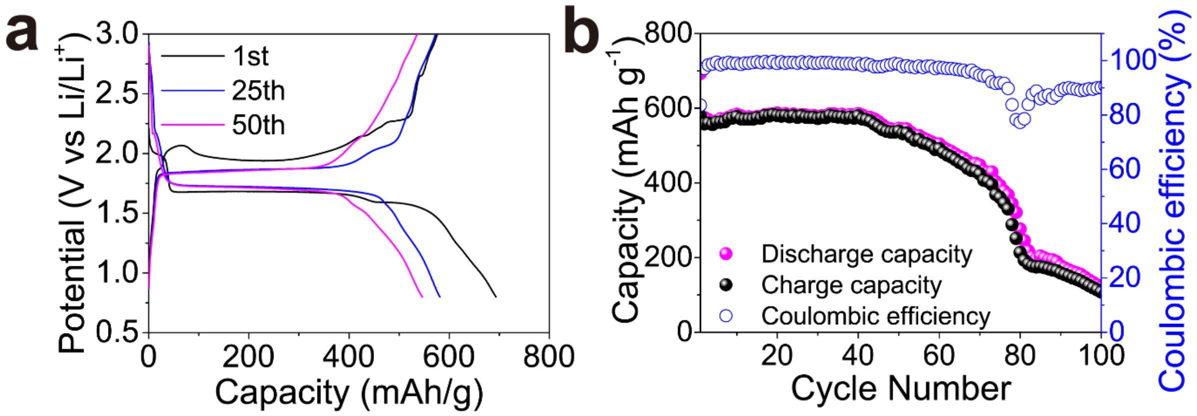

Figure S12. Electrochemical lithium storing performance of CuS. (a) Charge and discharge profiles and **(b)** cyclic performance of CuS between 0.8 V to 3 V at 0.2 C. For electrolyte, 1M lithium bis(trifluoromethanesulfonyl)imide in diglyme is used.

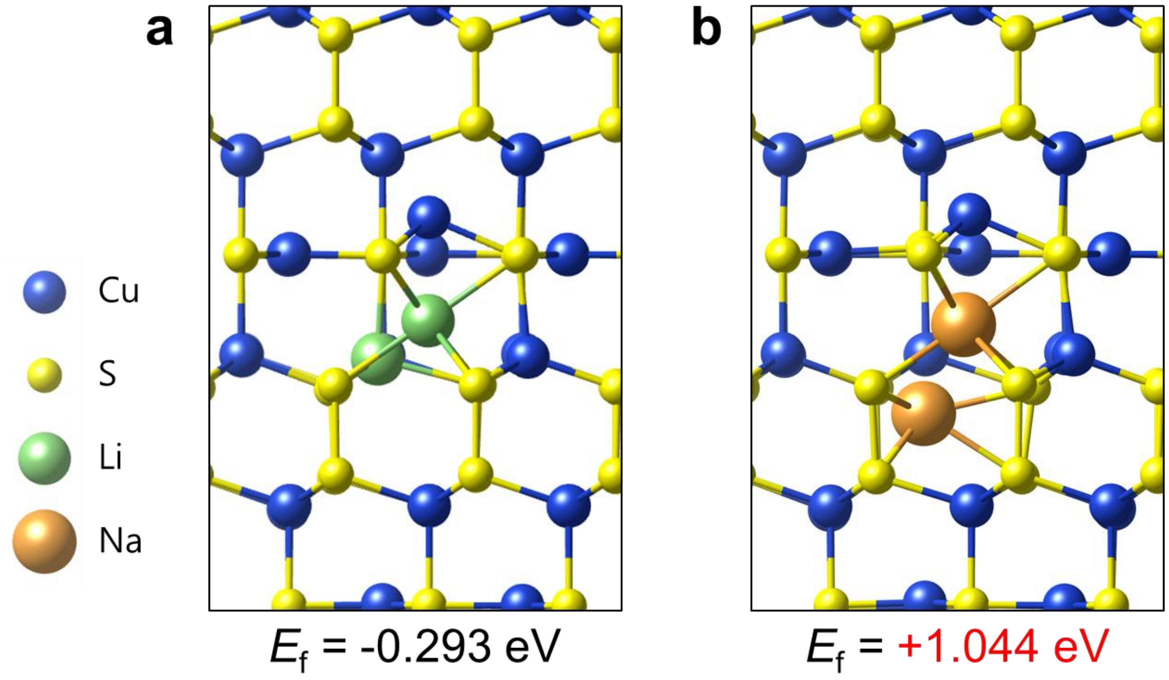

Figure S13. Defect formation energy for substituting Cu. Structures and formation energies (E_f) for **(a)** 2 Li substituting a Cu site and **(b)** 2 Na substituting a Cu site. Substituting Na ions is unstable and large Na ions tend to occupy interstitial sites rather than replace the Cu site, which induces formation of Na-Cu-S ternary system.

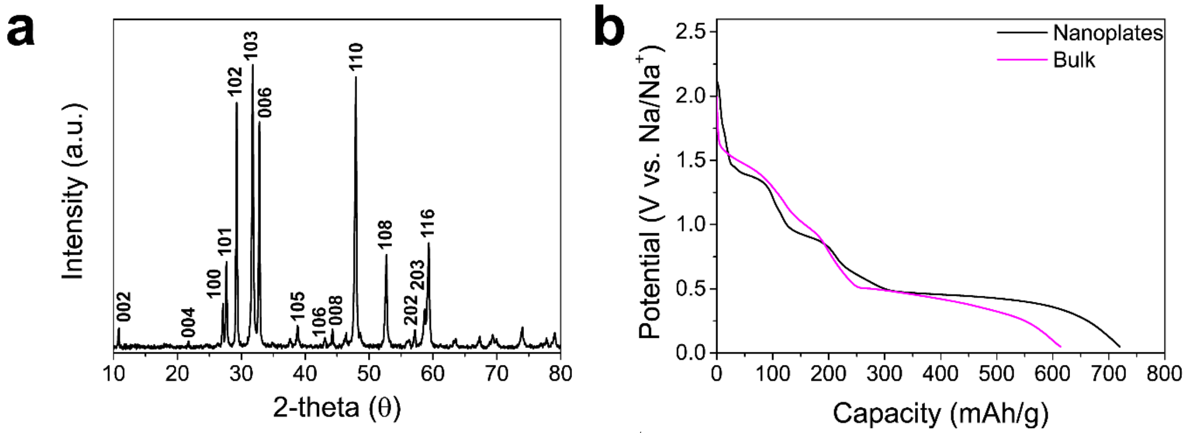

Figure S14. (a) X-ray diffraction (XRD) of bulk CuS. **(b)** 1st discharge profiles in CuS bulk and nanoplates at 0.2C and 1C, respectively.

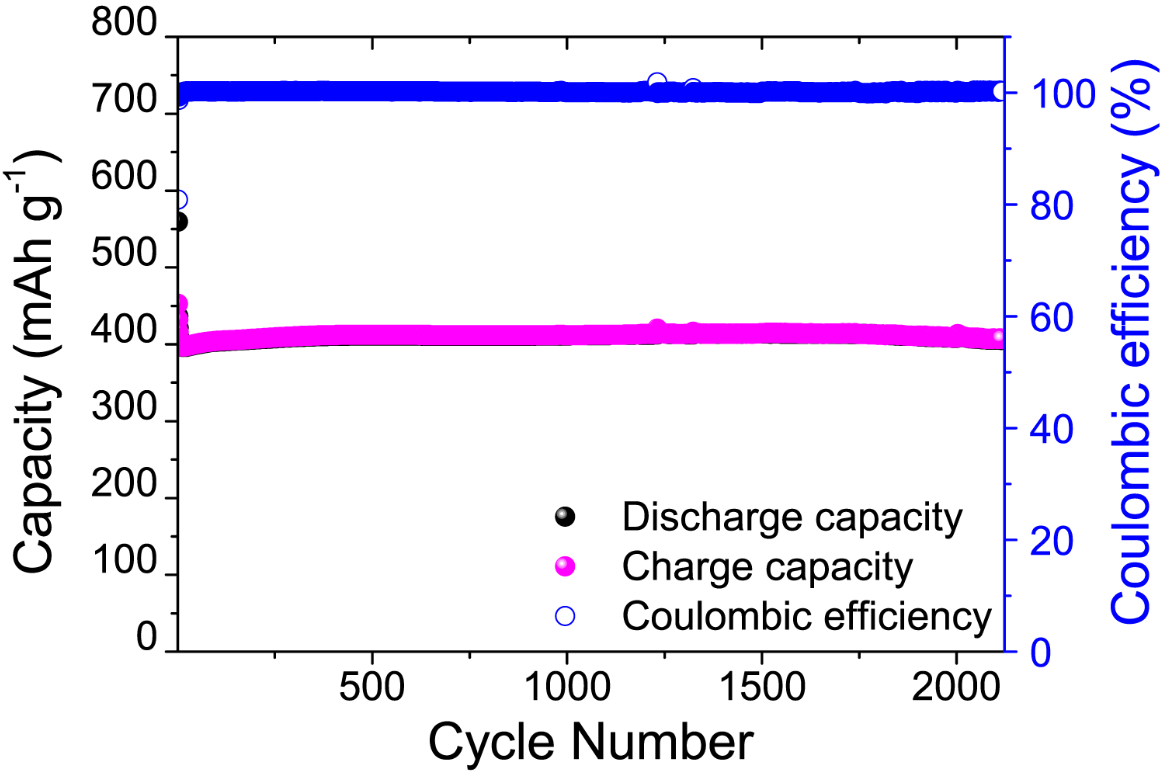

Figure S15. Cyclclic performance of bulk CuS at 1 C for 2120 cycles.

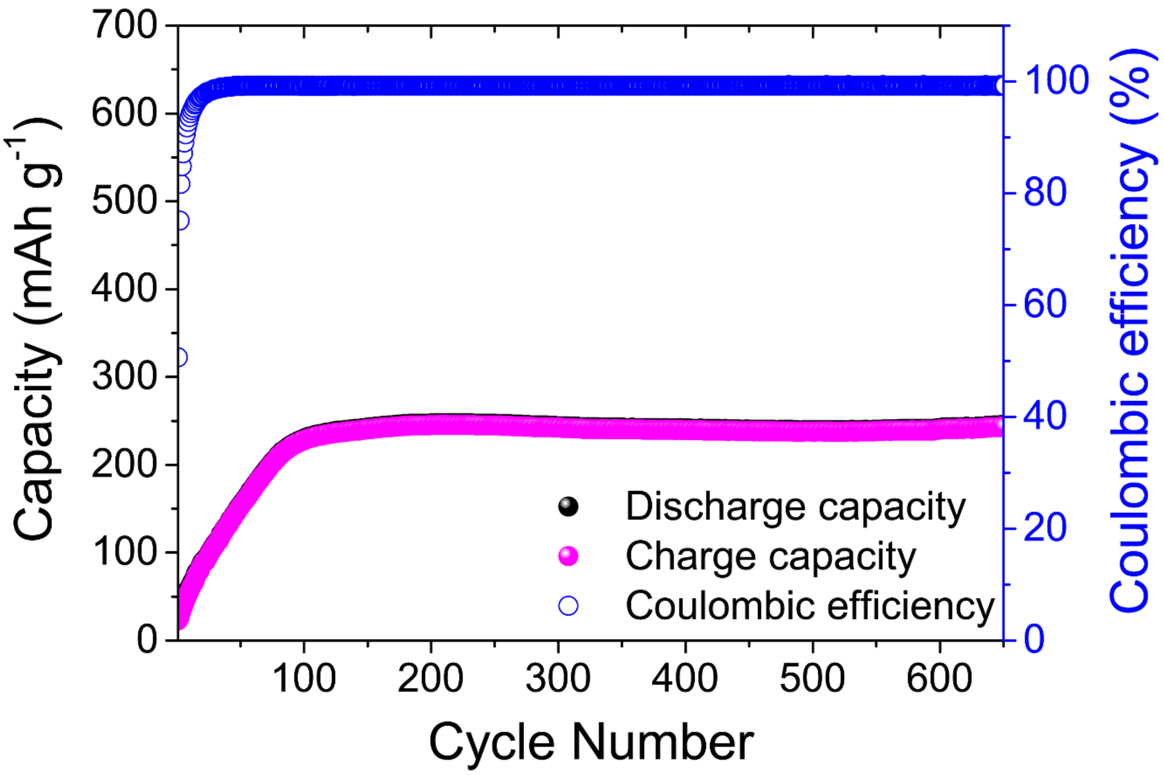

Figure S16. Cyclic performance of bulk CuS at 10 C.

Supplementary Method

Density functional theory (DFT) calculation: For DFT calculations, we use Vienna *Ab initio* Simulation Package (VASP)[1] and employ the generalized gradient approximation (GGA) for the exchange-correlation functional between electrons with 300 eV cut-off energy of the plane-wave basis. To calculate defect formation energy, we use 3×3×1 super-cell (108 atoms) of CuS unit-cell ($P6_3/mmc$) and 2×2×1 Monk-horst pack for the ***k***-point sampling. This setting ensures that energy and pressure is converged less than 0.01 eV/atom and 10 kbar, respectively. The formation energies of Li and Na substitutional defects at Cu site are calculated using the following equation:

$$E_f[M_{Cu}] = \frac{E[2M_{Cu}] - E[CuS] - 2 \cdot E[M]}{2}$$

where $E_f[M_{Cu}]$ is M substitutional defect on Cu site (M = Li, Na), $E[2M_{Cu}]$ is the total energy of super-cell with two M substituting one Cu, E[CuS] is the total energy of defect-free CuS super-cell, and $E[M]$ is the energy of metal M phase per ion. In all defect calculations, ions are fully relaxed that forces on ions are less than 0.02 eV/Å while lattice parameters are fixed from the fully-relaxed pristine structure.

Supplementary reference

[1] Kresse, G. and Hafner, J. Ab initio molecular dynamics for liquid metals. *Physical Review B* **1993**, *47*, 558-561.
